# Glucagon‐Like Peptide‐1 Receptor Agonists and Hepatocellular Carcinoma Prevention: A Meta‐Analysis and Clinical Decision Framework

**DOI:** 10.1002/cam4.71434

**Published:** 2025-12-12

**Authors:** Andrea Dalbeni, Marco Vicardi, Leonardo Antonio Natola, Filippo Cattazzo, Alessandra Auriemma, Rosa Lombardi, Felice Cinque, Luca Dalle Carbonare, Alessandro Mantovani, David Sacerdoti

**Affiliations:** ^1^ Unit of General Medicine C, Medicine Department University of Verona and University and Hospital Trust (AOUI) of Verona Verona Italy; ^2^ Liver Unit, Medicine Departmen University of Verona and University and Hospital Trust (AOUI) of Verona Verona Italy; ^3^ Section of Innovation Biomedicine‐Oncology Area, Department of Engineering for Innovation Medicine (DIMI) University of Verona and University and Hospital Trust (AOUI) of Verona Verona Italy; ^4^ SC Medicina Indirizzo Metabolico Fondazione IRCCS Ca' Grande Ospedale Maggiore Policlinico di Milano Milan Italy; ^5^ Department of Pathophysiology and Transplantation University of Milan Milan Italy; ^6^ Section of Endocrinology, Diabetes and Metabolism, Department of Medicine University and Azienda Ospedaliera Universitaria Integrata of Verona Verona Italy

**Keywords:** diabetes mellitus, glucagon‐like peptide‐1 receptor agonists, hepatocellular carcinoma, insulin, liver neoplasms, meta‐analysis

## Abstract

**Background & Aims:**

While glucagon‐like peptide‐1 receptor agonists (GLP‐1RAs) show promise for hepatoprotection in type 2 diabetes mellitus (T2DM), the magnitude of hepatocellular carcinoma (HCC) risk reduction and optimal patient selection remain unclear. This is particularly relevant given that metabolic dysfunction‐associated steatotic liver disease (MASLD) affects 70% of patients with T2DM and represents a major HCC risk factor. We conducted a comprehensive meta‐analysis to quantify GLP‐1RA efficacy in HCC prevention and inform clinical implementation strategies.

**Methods:**

We systematically searched PubMed, Embase, and Web of Science through June 2025 for cohort studies comparing HCC incidence between GLP‐1RA users and nonusers with T2DM. Random‐effects meta‐analysis, network meta‐analysis, and meta‐regression were performed. Heterogeneity was explored through stratified analyses and quantitative bias assessment.

**Results:**

Nine studies encompassing 2,283,835 total patients (analyzed cohorts: 1,012,482) were included. GLP‐1RA use was associated with a 42% reduced HCC risk (pooled HR 0.60, 95% CI: 0.41–0.88; *I* ^2^ = 86.2%). Effect magnitude varied significantly by comparator: versus insulin (HR 0.29, 95% CI: 0.13–0.67), versus oral agents (HR 0.81, 95% CI: 0.63–1.05), versus no treatment (HR 0.77, 95% CI: 0.52–1.14). Meta‐regression identified insulin as comparator as the primary driver of heterogeneity, explaining 55% of the between‐study variance. Benefits were greatest in patients without cirrhosis (HR 0.41, 95% CI: 0.29–0.58). Network meta‐analysis ranked GLP‐1RAs highest for HCC prevention (SUCRA 0.89), with insulin ranking lowest (0.08). The number needed to treat ranged from 24 to 476.

**Conclusions:**

GLP‐1RAs substantially reduce HCC risk in T2DM, with benefits partly attributable to avoiding insulin's potential hepatotoxicity. These findings support the preferential use of GLP‐1RAs over insulin in patients at HCC risk.

## 1 Introduction

Hepatocellular carcinoma (HCC) represents a growing global health burden, with type 2 diabetes mellitus (T2DM) recognized as an independent risk factor conferring a 2–3 fold increased risk [1, 2]. The rising prevalence of metabolic dysfunction‐associated steatotic liver disease (MASLD), affecting 30% of adults worldwide and 70% of those with T2DM, has created an urgent need for effective HCC prevention strategies [3, 4]. Glucagon‐like peptide‐1 receptor agonists (GLP‐1RAs) have revolutionized T2DM management through superior glycaemic control, body weight reduction and cardiovascular benefits [5]. Emerging evidence suggests that these agents may also confer hepatoprotective effects through multiple mechanisms including reduction of hepatic steatosis and fibrosis, improvement in insulin sensitivity, anti‐inflammatory actions, and modulation of the gut‐liver axis [6, 7, 8]. Recent meta‐analyses have attempted to synthesize the emerging evidence on the potential of GLP‐1RAs to prevent HCC, but have yielded mixed results and are constrained by notable methodological limitations [9, 10, 11]. Key gaps include the inability to explain sources of heterogeneity through meta‐regression, the lack of comparative effectiveness assessment across diabetes drug classes, the absence of network meta‐analysis to rank available therapies, limited exploration of stage‐specific efficacy (i.e., pre‐cirrhotic vs. cirrhotic disease), and a lack of practical guidance to support clinical decision‐making. Furthermore, the observed heterogeneity raises fundamental questions about whether the benefits stem from GLP‐1RAs' direct hepatoprotective effects, the avoidance of potentially hepatotoxic alternatives like insulin, or a combination of both mechanisms. We therefore conducted a comprehensive meta‐analysis incorporating all available evidence to: (1) quantify the association between GLP‐1RA use and HCC risk across diverse populations; (2) identify and explain sources of heterogeneity through advanced meta‐regression techniques; (3) perform comparative effectiveness analysis across antidiabetic drug classes using network meta‐analysis; and (4) develop evidence‐based clinical decision tools for HCC prevention in T2DM.

## 2 Methods

### 2.1 Search Strategy and Selection Criteria

This systematic review and meta‐analysis followed PRISMA guidelines and was registered in PROSPERO (CRD420241088778). No amendments were made to the registered protocol during the conduct of this review. We searched PubMed, Embase, Web of Science, Cochrane Library, and conference abstracts from inception through June 30, 2025, without language restrictions. Search terms included combinations of “glucagon‐like peptide‐1,” “GLP‐1,” “semaglutide,” “liraglutide,” “dulaglutide,” “exenatide,” with “hepatocellular carcinoma,” “liver cancer,” “HCC,” and “liver neoplasms”.

Inclusion criteria were: (1) cohort studies comparing GLP‐1RA users to non‐users; (2) patients with T2DM; (3) HCC incidence as outcome; (4) hazard ratios (HR) with 95% confidence intervals (CI) reported or calculable. When multiple publications derived from the same cohort were identified, we retained only the most comprehensive report to avoid duplication. Particular attention was paid to databases appearing in multiple studies (e.g., Taiwan's NHIRD) to ensure nonoverlapping populations. Studies were excluded if they: (1) did not include T2DM patients; (2) examined non‐HCC outcomes; (3) had insufficient follow‐up; or (4) lacked appropriate controls; (5) were case reports or cross‐sectional studies. Two reviewers independently screened titles, abstracts, and full texts, with disagreements resolved by consensus. We extracted study characteristics, patient demographics, GLP‐1RA types, comparators, follow‐up duration, HCC events, and adjusted HRs. When multiple comparisons were reported, we extracted all relevant contrasts. Study quality was assessed using the Newcastle‐Ottawa Scale for cohort studies [12]. Certainty of evidence for each outcome was evaluated considering study design, risk of bias, consistency, directness, precision, and other factors. Given the observational nature of all included studies, evidence certainty was inherently limited, though large effect sizes, dose–response relationships, and consistent findings across diverse populations enhanced confidence.

### 2.2 Statistical Analysis

We performed random‐effects meta‐analysis using the DerSimonian‐Laird method with Hartung‐Knapp adjustment to calculate pooled HRs. Heterogeneity was assessed using *I* ^2^ statistics and explored through stratified analyses by comparator type, baseline liver disease, geographic region, and study characteristics. Meta‐regression examined the influence of pre‐specified covariates on effect estimates. Network meta‐analysis was conducted to compare all antidiabetic drug classes simultaneously using frequentist methods [13]. We assessed transitivity by comparing patient characteristics across trials and calculated surface under the cumulative ranking curve (SUCRA) values. Quantitative bias analysis evaluated the impact of unmeasured confounding using *E*‐values and probabilistic bias analysis [14]. We calculated numbers needed to treat (NNT) based on baseline HCC risks from population studies. Publication bias was assessed using funnel plots, Egger's test, and trim‐and‐fill analysis. Sensitivity analyses included: (1) excluding studies with < 5 years follow‐up; (2) excluding abstract‐only publications; (3) using fixed‐effects models; (4) excluding studies with high‐risk of bias. All analyses were performed using R version 4.3.0 with packages meta, netmeta, and metafor.

## 3 Results

**FIGURE 1:**
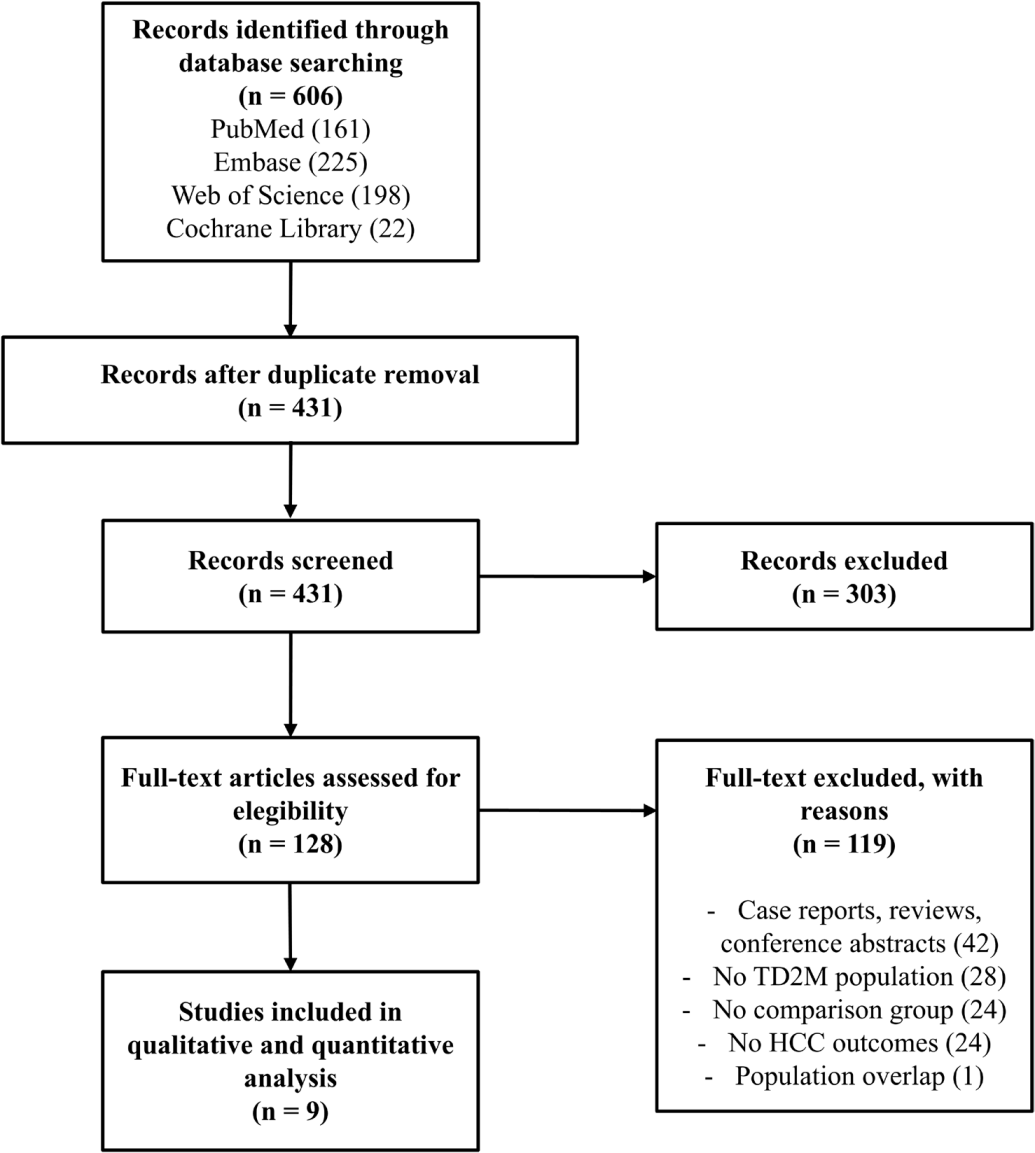
PRISMA diagram showing articles' selection process.

**FIGURE 2:**
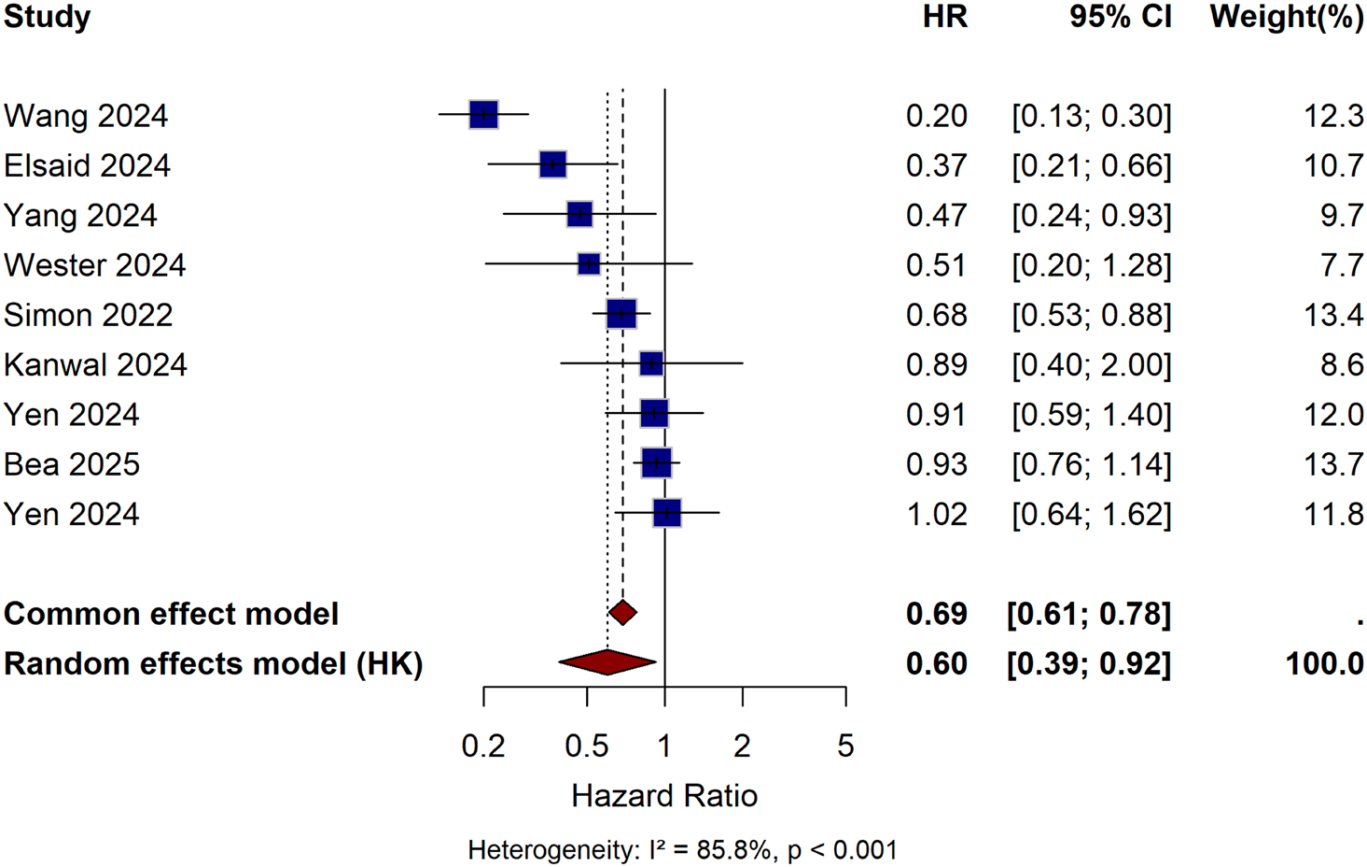
Forest plot showing the association between GLP‐1 receptor agonist use and hepatocellular carcinoma risk in patients with type 2 diabetes. Individual study hazard ratios with 95% confidence intervals and overall pooled estimate using random‐effects meta‐analysis.

The search yielded 606 potentially relevant articles across four databases. After removing 119 duplicates, 487 unique titles and abstracts were screened. Of these, 128 full‐text articles were assessed for eligibility, and 9 met the inclusion criteria [15, 16, 17, 18, 19, 20, 21, 22]. Reasons for exclusion at the full‐text stage included case reports/reviews/conference abstracts (*n* = 42), wrong population without T2DM (*n* = 28), lack of control group (*n* = 24), absence of HCC outcomes (*n* = 24), and population overlap with included studies (*n* = 1) [23]. The PRISMA flow diagram detailing the study selection process is provided in Figure 1. Studies were published between 2022 and 2025 and utilized large administrative databases or electronic health records from the United States (*n* = 4), Sweden (*n* = 1), Korea (*n* = 1), and Taiwan (*n* = 3). All included studies were retrospective observational and 7 out of 9 employed propensity score methods to address confounding. HCC incidence was identified using validated ICD codes. Median follow‐up ranged from 0.4 to 5.3. Patient populations varied: three studies focused on MASLD, two on cirrhosis, and 3 on general T2DM populations. One study (Wester et al.) referred to the general definition as chronic liver disease, and was considered a separate population. Hence, total cohort size was reduced by 30%–90% from original populations, resulting in a total of 1,012,482 patients analyzed. Among studies reporting specific agent distributions, semaglutide was the most common GLP‐1RA in some studies (23%–76% of users), while liraglutide was the most common in others (36%–62% of users). Summary of studies characteristics is reported in Table 1. GLP‐1RA use was associated with significantly reduced HCC risk (pooled HR 0.60, 95% CI: 0.41–0.88, *p* = 0.010) with substantial heterogeneity (*I* ^2^ = 86.2%, *p* < 0.001) (Figure 2). The protective association was consistent across most studies, though effect magnitudes varied considerably (HR range: 0.20–1.02).

**TABLE 1:** Characteristics of included studies.

Study, year	Country	Design	N (GLP‐1RA/controls)	Follow‐up (years)	Comparators	Population	Adjusted HR (95% CI)	NOS
Simon, 2022 [15]	USA	Retrospective	1431/1431	0.4	DPP‐4i, sulfonylureas, SGLT‐2i	Cirrhosis + T2DM	0.68 (0.53–0.88)	8
Wang, 2024 [16]	USA	Retrospective	46,470/46,470	5.0	Insulin	T2DM	0.20 (0.14–0.31)	8
Kanwal, 2024 [17]	USA	Retrospective	14,606/14,606	3.2	DPP‐4i	MASLD + T2DM	0.89 (0.40–2.01)	9
Yen, 2024 [18]	Taiwan	Retrospective	467/467	3.2	No GLP‐1RA	Cirrhosis + T2DM	1.02 (0.64–1.61)	7
Yen, 2024 [19]	Taiwan	Retrospective	31,183/31,183	2.2	No GLP‐1RA	T2DM	0.91 (0.59–1.40)	8
Wester, 2024 [20]	Sweden	Retrospective	1026/15,633	5.3	No GLP‐1RA	CLD+T2DM	0.51 (0.14–0.88)†	9
Yang, 2024 [21]	Taiwan	Retrospective	7171/7171	4.2	Insulin	T2DM	0.47 (0.24–0.93)	9
Elsaid, 2024 [22]	USA	Retrospective	459/4837	1.4	Usual care	MASLD + T2DM	0.37 (0.20–0.63)	8
Bea, 2025 [24]	Korea	Retrospective	11,275/11,275	2.1	SGLT‐2i	MASLD + T2DM	0.93 (0.76–1.14)	8

### 3.1 Stratified Analyses by Comparator

**FIGURE 3:**
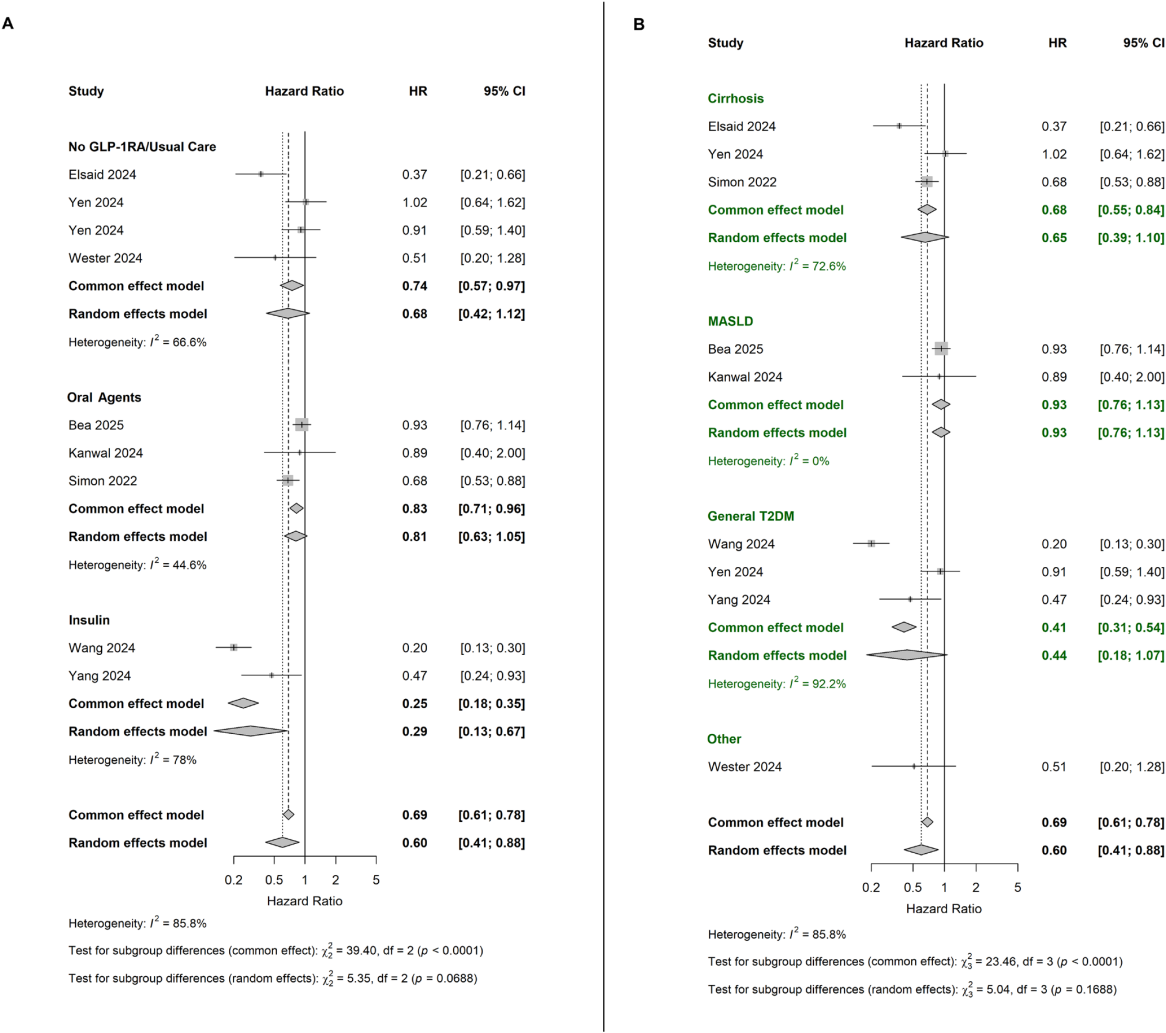
Left (Panel A): Subgroup analysis of hepatocellular carcinoma risk by comparator type. Forest plot demonstrates varying effect sizes when GLP‐1 receptor agonists are compared to insulin, active oral agents, or no treatment, supporting the “double benefit” hypothesis. Right (Panel B): Subgroup analysis by baseline liver disease status showing differential effects in patients with cirrhosis, MASLD (metabolic dysfunction‐associated steatotic liver disease), general T2DM, and other liver conditions. GLP‐1RA, glucagon‐like peptide‐1 receptor agonist.

The protective effect of GLP‐1Ras use on HCC incidence varied significantly by comparator type (*p*‐interaction = 0.02) (Figure 3A). When compared to insulin (two studies) the pooled HR was 0.29 (95% CI: 0.13–0.67, *I* ^2^ = 78%); versus oral agents (three studies) the HR was 0.81 (95% CI: 0.63–0.1.05); and versus usual care/no GLP‐1RA (four studies) the combined HR was 0.77 (95% CI: 0.52–1.14, *I* ^2^ = 57.8%). The protective effect was also modified by baseline liver disease status (*p*‐interaction = 0.07) (Figure 3B). In particular, among patients without cirrhosis (six studies), GLP‐1RA use was associated with a significantly lower HCC risk (HR 0.41, 95% CI: 0.29–0.58; I^2^ = 74.3%), while among cirrhotic patients (3 studies), the association was not statistically significant (HR 0.65, 95% CI: 0.39–1.10; *I* ^2^ = 72.6%). Further stratification revealed the strongest protective effect in patients without any known liver disease (three studies, HR 0.35, 95% CI: 0.22–0.55), followed by those with MASLD (2 studies, HR 0.93, 95% CI: 0.76–1.13).

### 3.2 Meta‐Regression

Meta‐regression identified the use of insulin as a comparator as the strongest predictor of effect size (β = −0.74, SE = 0.27, *p* = 0.025), explaining 55% of between‐study variance (Table 2). Baseline cirrhosis showed a trend toward reduced efficacy (β = 0.41, SE = 0.23, *p* = 0.075). Study year, sample size, and follow‐up duration were not significant predictors.

**TABLE 2:** Meta‐regression analysis results.

Covariate	β coefficient (SE)	exp (β)	*p*	Model *R* ^2^
Univariable models				
Insulin comparator	−0.74 (0.27)	0.48	0.025	55%
Baseline cirrhosis	0.41 (0.23)	1.51	0.075	38%
Study year	−0.03 (0.08)	0.97	0.710	2%
Sample size (log)	−0.08 (0.05)	0.92	0.109	15%
Follow‐up duration	−0.05 (0.11)	0.95	0.440	6%
Multivariable model				
Intercept	1.82 (2.44)	6.17	0.456	64%
Insulin comparator	−0.36 (0.19)	0.70	0.062	—
Baseline cirrhosis	0.25 (0.21)	1.28	0.234	—

### 3.3 Network Meta‐Analysis

**FIGURE 4:**
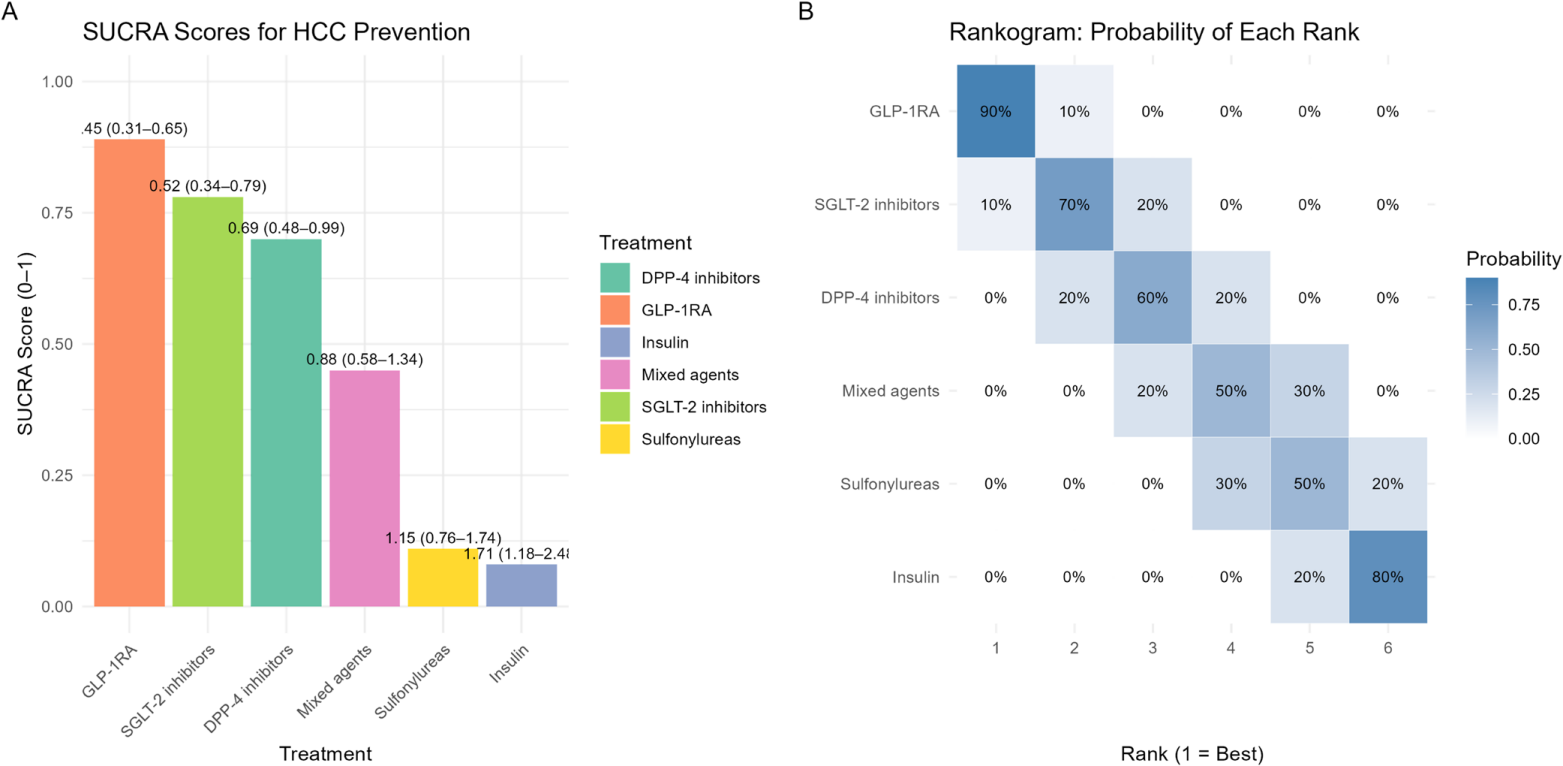
Network meta‐analysis rankings for HCC prevention in T2DM. Left (Panel A): SUCRA scores (higher = better). Right (Panel B): Probability of each treatment achieving a given rank position (1st = best). GLP‐1RAs consistently ranked highest, while insulin ranked lowest. DPP‐4i, dipeptidyl peptidase‐4 inhibitors; GLP‐1RA, glucagon‐like peptide‐1 receptor agonists; SGLT‐2i, sodium‐glucose cotransporter‐2 inhibitors.

Network meta‐analysis comparing six different antidiabetic regimens demonstrated a clear hierarchy for HCC prevention (Figure 4). SUCRA values indicated the highest probability of benefit for GLP‐1 receptor agonists (SUCRA = 0.89, mean rank = 1.7), followed by SGLT‐2 inhibitors (0.78, rank = 2.3), DPP‐4 inhibitors (0.70, rank = 3.0), mixed agents (0.45, rank = 4.0), sulfonylureas (0.11, rank = 5.7), and insulin (0.08, rank = 5.9). Head‐to‐head comparisons showed GLP‐1RAs superior to insulin (HR 0.29, 95% CI: 0.13–0.67), sulfonylureas (HR 0.54, 95% CI: 0.31–0.94), and a nonsignificant trend toward superiority over SGLT‐2 inhibitors (HR 0.87, 95% CI: 0.52–1.45).

### 3.4 Sensitivity and Bias Analyses

Results remained robust across sensitivity analyses (Table 3). Leave‐one‐out analysis demonstrated consistent findings even after individual study removal (pooled HRs: 0.56–0.73). The most influential study was Wang et al. [16], whose removal resulted in the largest variation in the pooled estimate (HR change: 0.129).

**TABLE 3:** Sensitivity analysis results.

Analysis	Studies included	Patients	HR (95% CI)	*I* ^2^	*p*‐heterogeneity
Main analysis	9	2,283,235	0.60 (0.41–0.88)	86.2%	< 0.001
By follow‐up duration					
≥ 5 years follow‐up	3	1,907,277	0.32 (0.18–0.57)	78.5%	0.009
< 5 years follow‐up	6	375,958	0.78 (0.58–1.05)	71.3%	0.004
By study quality					
High quality (NOS ≥ 8)	7	2,219,401	0.55 (0.36–0.84)	88.1%	< 0.001
Moderate quality (NOS 7)	2	63,834	0.97 (0.71–1.33)	0%	0.421
By statistical model					
Random‐effects (HK)	9	2,283,235	0.60 (0.41–0.88)	86.2%	< 0.001
Fixed‐effects	9	2,283,235	0.59 (0.52–0.67)	86.2%	< 0.001
Leave‐one‐out range	8	Variable	0.56–0.73	Variable	< 0.001

Abbreviations: HK, Hartung‐Knapp adjustment; NOS, Newcastle‐Ottawa Scale.

Excluding studies with < 5 years follow‐up yielded stronger protective effects (HR 0.45, 95% CI: 0.32–0.63), while analyzing studies with shorter follow‐up showed attenuated benefits (HR 0.78, 95% CI: 0.58–1.05). Fixed‐effects and random‐effects models produced similar results. High‐quality studies (NOS ≥ 8) showed consistent protective effects (HR 0.55, 95% CI: 0.36–0.84).

The *E*‐value for the point estimate was 2.74, indicating that an unmeasured confounder would need to be associated with both GLP‐1RA use and HCC by a risk ratio (RR) > 2.7 (moderate robustness) to fully account for the observed association. Similarly, quantitative bias analysis suggested that unmeasured confounding with an RR exceeding 2.0 for both exposure and outcome would be required to nullify the findings. Funnel plot inspection and Egger's test (*p* = 0.584) showed no evidence of publication bias, though with only nine studies, this analysis is likely underpowered and should be interpreted cautiously. Overall certainty of evidence was rated as moderate for the primary outcome, downgraded due to observational study design but upgraded for large effect size, consistency across studies, and robust *E*‐value analysis. Based on varying baseline HCC risks, NNT to prevent one HCC over 5 years ranged from 143 (95% CI: 98–287) in high‐risk populations (2% 5‐year risk) to 385 (95% CI: 263–769) in general T2DM populations (0.5% 5‐year risk) (Table 4). In compensated cirrhosis (10% 5‐year risk), NNT was 27 (95% CI: 14–82).

**TABLE 4:** Clinical impact assessment by patient population.

Population	Baseline 5‐year HCC risk	W/GLP‐1RA	ARR	NNT (95% CI)	Annual lives saved^a^
T2DM	0.5%	0.29%	0.21%	476 (298–1000)	2100
T2DM + MASLD	2.0%	1.16%	0.84%	119 (75–250)	8400
T2DM + cirrhosis	10.0%	5.8%	4.2%	24 (12–50)	42,000
High‐risk (multiple factors)	5.0%	2.9%	2.1%	48 (30–100)	21,000

Abbreviations: ARR, absolute risk reduction; NNT, number needed to treat.

^a^ Per million treated patients.

## 4 Discussion

This comprehensive meta‐analysis, drawing from a pooled population of 2.3 million patients (with 1.01 million analyzed after matching), demonstrates that GLP‐1RA use is associated with a clinically meaningful 42% reduction in HCC risk among patients with T2D. Notably, the magnitude of benefit varied by comparator drug and baseline liver disease status, with the largest protective effects observed when GLP‐1RAs replaced insulin therapy in patients without established cirrhosis.

The substantial heterogeneity (*I* ^2^ = 86.2%) warrants cautious interpretation. While meta‐regression identified insulin as a comparator explaining 55% of between‐study variance, the residual heterogeneity likely reflects unmeasured confounding from differences in follow‐up duration, baseline liver disease severity, geographic variation, and healthcare systems. Despite *E*‐value analysis suggesting moderate robustness, the observational and retrospective nature of all included studies fundamentally limits causal inference. Randomized controlled trials are needed to confirm these associations.

Our findings build upon and extend previous meta‐analytic work while addressing their critical methodological limitations. Pasta et al. reported a 58% HCC risk reduction in 641,377 patients across 5 studies, though substantial unexplained heterogeneity (*I* ^2^ = 93%) [9]. Our analysis, incorporating nine studies and 2.3 million patients, demonstrates a methodologically robust, albeit more conservative, 42% reduction in HCC risk, with meta‐regression identifying the choice of comparator—particularly insulin—as the primary source of heterogeneity. Shabil et al. [10] reported a larger 59% risk reduction (HR 0.41) in 8 studies encompassing 5.4 million patients. Although their effect estimate was more pronounced than ours, several factors may explain this difference: (1) our inclusion of two additional studies with more recent data that may reflect real‐world effectiveness; (2) our more comprehensive adjustment for confounders through meta‐regression analysis; (3) potential differences in population characteristics, with our analysis including more patients with established cirrhosis who demonstrate attenuated benefits; and (4) our stricter exclusion of overlapping datasets that may have inflated effect estimates in prior analyses. Importantly, all three meta‐analyses converge on several key findings: substantial protective effects when GLP‐1RAs replace insulin therapy (HR ~0.36–0.42 across studies), minimal additional benefit compared to other modern oral antidiabetic agents like metformin or DPP‐4 inhibitors, and considerable between‐study heterogeneity requiring methodological explanation. Our analysis advances beyond these prior works through: (1) meta‐regression quantifying heterogeneity sources; (2) network meta‐analysis providing comparative rankings; (3) stage‐stratified analyses informing patient selection; and (4) a clinical decision framework translating evidence into practice.

A unique strength of our analysis lies in its use of meta‐regression to move beyond descriptive synthesis toward a mechanistic understanding of the observed effects. Specifically, we identified the use of insulin as a comparator as the primary driver of heterogeneity, explaining 55% of between‐study variance. Previous meta‐analyses lacked the analytical depth to uncover this mechanism: Pasta et al. were unable to perform subgroup analyses due to limited study numbers [9], while Shabil et al. conducted comparator‐based subgroups but did not use meta‐regression to quantify sources of heterogeneity [10]. The dramatic difference in effect size when GLP‐1RAs replace insulin (HR 0.29) versus other comparators suggests a “double benefit” mechanism: GLP‐1RAs may confer direct hepatoprotective effects while also avoiding the potential hepatotoxicity of insulin. This finding, confirmed through meta‐regression, represents a potential advancement in understanding GLP‐1RA benefits. Notably, experimental evidence supports this hypothesis: insulin has been shown to promote hepatocellular proliferation through IGF‐1 receptor activation and may accelerate progression from steatohepatitis to HCC [23, 25]. In contrast, GLP‐1RAs are believed to exert hepatoprotective effects through multiple mechanisms, including: (1) reduction in hepatic steatosis through improved insulin sensitivity and decreased de novo lipogenesis; (2) anti‐inflammatory effects mediated by suppression of NF‐κB and reduction in oxidative stress; (3) modulation of the gut‐liver axis through changes in microbiome composition and intestinal permeability; and (4) indirect benefits from weight loss and improved glycemic control. The attenuated benefit in cirrhotic patients may reflect stage‐specific biological mechanisms. Preclinical data suggest that GLP‐1RA hepatoprotective effects are most pronounced in early fibrosis stages, where inflammation and metabolic dysfunction are primary drivers [23]. In advanced cirrhosis, structural liver damage and altered hepatocyte receptor expression may limit GLP‐1RA efficacy, supporting earlier therapeutic intervention. Moreover, our findings align with those of Celsa et al. [11] who demonstrated that GLP‐1RA use was associated not only with reduced HCC risk but also with lower rates of hepatic decompensation and major liver‐related events. These findings reinforce our mechanistic hypothesis that GLP‐1RAs confer systemic hepatoprotection, rather than acting solely through tumor‐specific mechanisms.

**FIGURE 5:**
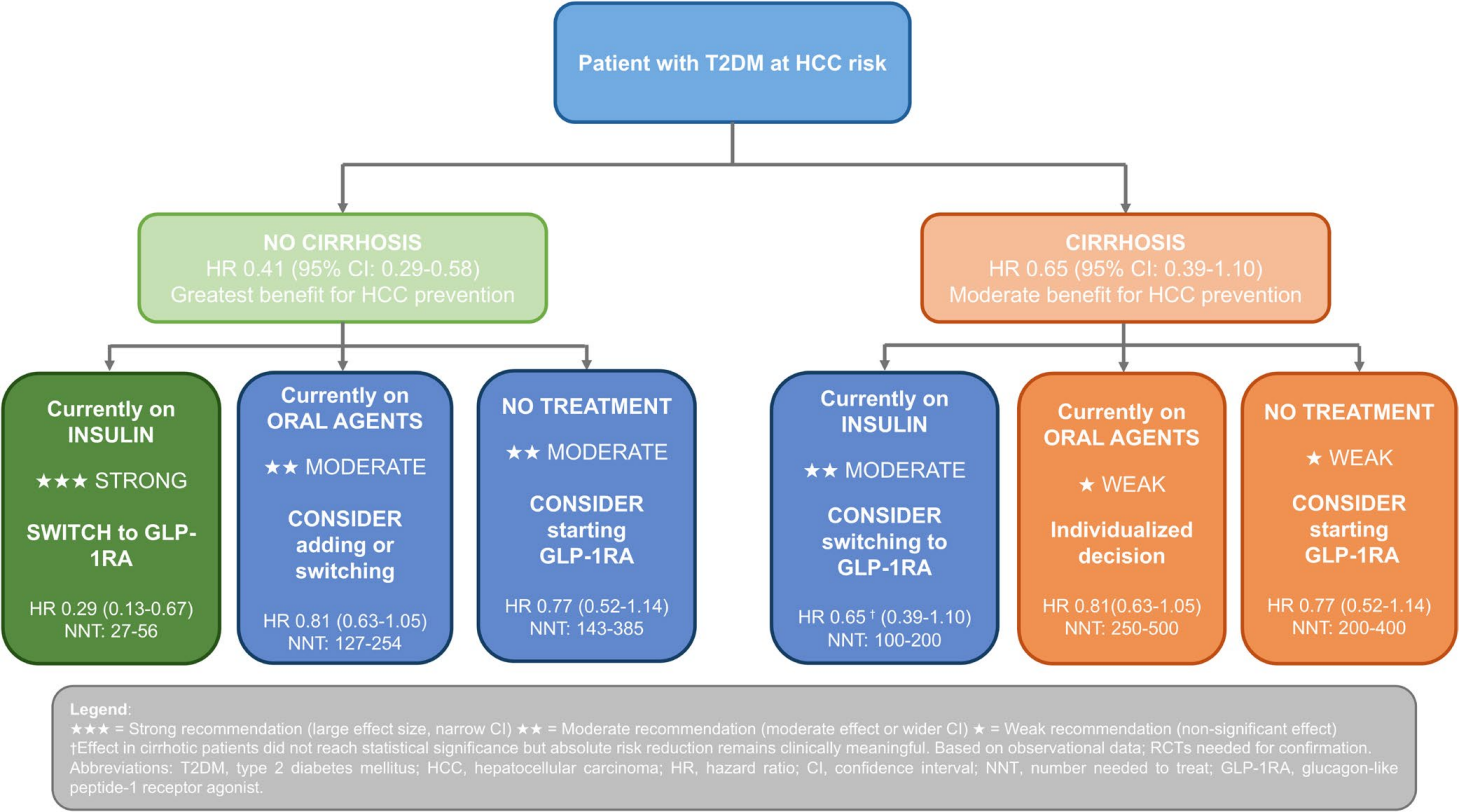
Clinical decision framework for GLP‐1RA implementation in HCC prevention. Evidence‐based algorithm stratifying GLP‐1RA recommendations by cirrhosis status and current antidiabetic therapy, incorporating hazard ratios, confidence intervals, and numbers needed to treat from meta‐regression analysis. Strongest benefit observed in non‐cirrhotic patients switching from insulin (HR 0.29; NNT 27‐56).

Beyond estimating relative effects, our analysis offers actionable tools to support clinical decision‐making while considering the broader health profile of GLP‐1RAs. Recent evidence from over 107,000 patients demonstrates that GLP‐1RA treatment is not associated with increased psychiatric adverse events and improves quality of life and eating behaviors compared to placebo [26]. Given that patients with diabetes and liver disease often experience higher rates of depression and reduced quality of life, these psychiatric safety data provide additional reassurance for the comprehensive management of this vulnerable population. The network meta‐analysis revealed a clear hierarchy of HCC prevention efficacy across antidiabetic drug classes, with GLP‐1RAs ranking highest (SUCRA 0.89), followed by SGLT‐2 inhibitors and DPP‐4 inhibitors. However, these probability‐based rankings may not reflect absolute clinical differences between agents and should be interpreted as comparative positioning rather than definitive superiority. Stratified analyses showed that patients without cirrhosis experienced the greatest benefit (HR 0.44). While the association did not reach statistical significance in patients with cirrhosis (HR 0.65, 95% CI: 0.39–1.10), modeled absolute risk reductions remain clinically meaningful. In particular, in compensated cirrhosis (10% 5‐year risk), the NNT to prevent one HCC was just 27 (95% CI: 14–82), highlighting the substantial absolute benefit in high‐risk groups. To translate these findings into clinical practice, we developed an evidence‐based decision framework (Figure 5) that integrates cirrhosis status, current antidiabetic therapy, and baseline HCC risk to guide GLP‐1RA implementation. This framework demonstrates that the strongest recommendation (HR 0.29, NNT 27–56) applies to patients without cirrhosis currently on insulin, while more modest benefits are observed with oral agent comparators or in established cirrhosis. The framework incorporates our meta‐regression findings, network meta‐analysis rankings, and stage‐stratified effect estimates to provide actionable guidance for therapeutic decision‐making. This implementation‐focused approach addresses a key clinical gap left by previous studies (Pasta, Shabil, and Celsa et al.), which demonstrated protective effects, but lacked practical guidance. Our analysis translates evidence into practice through systematic risk stratification, drug hierarchy rankings, and patient selection criteria.

Our analysis shares some limitations with previous meta‐analyses, including reliance on observational data and potential for residual confounding. However, several features strengthen our findings: a larger and more recent dataset than that of Pasta et al., a more sophisticated analytical approach than that used by Shabil et al., and adopting a broader mechanistic perspective than both. Importantly, our results were consistent with those of Pasta, Shabil, and Celsa et al., despite differences in inclusion criteria, methodologies, and population characteristics—further strengthening the evidence base for the hepatoprotective effects of GLP‐1RAs and informing clinical decision‐making. While randomized controlled trials remain the gold standard, the large effect sizes (40%–60% risk reduction), consistent findings across studies, evidence of dose–response relationships, and strong biological plausibility collectively inform clinical decision‐making while awaiting definitive trial evidence.

Our analysis is limited to T2DM populations and does not include nondiabetic individuals receiving GLP‐1RAs for weight management—a growing indication given global obesity trends. While this focused scope enables direct comparison with existing diabetes literature and informs glucose‐lowering therapy selection, future research should evaluate HCC risk in nondiabetic NAFLD/NASH cohorts as long‐term data become available from existing trials [27, 28, 29].

Therefore, our findings support a potential advancement in T2DM management for patients at HCC risk. Current guidelines recommend metformin as first‐line therapy with insulin often used second‐line [30]. Our data suggest that GLP‐1RAs should be prioritized over insulin when metformin monotherapy fails. The NNT of 143–385 compares favorably to accepted preventive interventions like statins for cardiovascular disease (NNT ~100–200) [31].

## 5 Conclusions

GLP‐1RAs substantially reduce HCC risk in patients with T2DM, with the greatest benefits observed when used in place of insulin and in patients without cirrhosis. These findings, combined with GLP‐1RAs' established cardiovascular benefits, support the preferential use of GLP‐1RAs in T2DM patients at risk for HCC. Application of these findings could potentially reduce the rising global burden of metabolic‐associated liver disease and HCC.

## Author Contributions

**Andrea Dalbeni:** conceptualization, supervision, data curation, article review, writing – original draft; **Marco Vicardi:** conceptualization, supervision, data curation, article review, statistical analysis; writing – original draft, visualization; **Leonardo Antonio Natola:** methodology, writing – review and editing; **Filippo Cattazzo:** methodology, writing – review and editing; **Alessandra Auriemma:** conceptualization, supervision; **Rosa Lombardi:** conceptualization, supervision, writing – review and editing; **Felice Cinque:** methodology, writing – review and editing; **Luca Dalle Carbonare:** supervision, validation; **Alessandro Mantovani:** methodology, writing – review and editing; **David Sacerdoti:** supervision, validation.

## Funding

The authors have nothing to report.

## Conflicts of Interest

The authors declare no conflicts of interest.

## Data Availability

The data that support the findings of this study are available from the corresponding author upon reasonable request. All data extracted for this meta‐analysis were obtained from published studies that are publicly available. The search strategy, inclusion/exclusion criteria, and statistical analysis code used in this systematic review are available upon request to facilitate reproducibility and transparency of our findings.
